# Bone Mineral Density of Femur and Lumbar and the Relation between Fat Mass and Lean Mass of Adolescents: Based on Korea National Health and Nutrition Examination Survey (KNHNES) from 2008 to 2011

**DOI:** 10.3390/ijerph17124471

**Published:** 2020-06-22

**Authors:** Aram Kim, Seunghui Baek, Seyeon Park, Jieun Shin

**Affiliations:** 1 Department of Sport Leisure, Sungshin Women’s University, Bomun-ro 34da-gil, Seongbuk-gu, Seoul 02844, Korea; ramii0021@gmail.com; 2 Department of Health Exercise Management, Sungshin Women’s University, Bomun-ro 34da-gil, Seongbuk-gu, Seoul 02844, Korea; sh100@sungshin.ac.kr; 3 Department of Nursing Health Institute of Technology, 21 Chungjeong-ro, Dong-gu, Daejeon 34504, Korea; sy_park@hit.ac.kr; 4 Liberal Arts, Woosuk University, 443, Samnye-ro, Samnye-eup, Wanju-gun, Jeollabuk-do 55338, Korea

**Keywords:** bone mineral density, femur, lumbar, adolescents, big data

## Abstract

It is most important to reach the maximum bone density in the childhood period to prevent developing osteoporosis; it is widely known that increased body weight has a positive correlation with bone density and that even though both the fat mass and lean mass have a significant impact on bone density, the latter mass has more importance for adults. Therefore, the study analyzed to identify the relationship between bone density and both fat mass and lean mass of Korean adolescents. Subjects were chosen among 21,303 people from the Korea National Health and Nutrition Examination Survey (KNHNES) between 2008 and 2011 that took a bone density checkup; as a result, 1454 teenagers aged between 12 and 18 were selected. Data analysis was performed in SAS ver. 9.4 (SAS Institute Inc., Cary, NC, USA) following the KNHNES and the weighted complex sample analysis was conducted; body fat mass and lean mass were divided into quintile groups, and to figure out the differences in bone density that were analyzed in six models adjusted by body weight (kg) and walking (yes/no), muscle strengthening exercises (yes/no), nutrition (intake of ca (g), and serum vitamin D (ng/mL)). Then, the generalized linear model (GLM) and trend test were conducted for each gender with a significance level of 0.05. The bone density differences of fat mass and lean mass were analyzed. The result of Model 6 considering all correction variables is as follows; in the case of male adolescents, the total femur and lumbar spine showed a significant difference (F = 13.120, *p* < 0.001; F = 12.900, *p* < 0.001) for fat mass, and the trend test showed that the figures significantly decreased (β = −0.030, *p* < 0.001; −0.035, *p* < 0.001). Meanwhile, for lean mass, the total femur and lumbar spine had a significant difference (F = 16.740, *p* < 0.001; F = 20.590, *p* < 0.001) too, but the trend test showed a significant increase (β = 0.054, *p* < 0.001; 0.057, *p* < 0.001). In the case of female adolescents, the lumbar spine (F = 3.600, *p* < 0.05) for lean mass showed a significant difference, and it also significantly rose in the trend test too (β = 0.020, *p* < 0.01). To sum up the results, for male adolescents, the bone density differences for fat mass (FM) and lean mass (LM) all had significant differences, but for female adolescents, only the lumbar spine for LM showed such a result. Meanwhile, both genders showed that LM had a more positive impact on bone density than FM.

## 1. Introduction

The morbidity rate and mortality rate of osteoporotic fracture are in a correlation [1]. According to research, the number of patients across the world with a hip fracture will increase to 6.3 million by 2050 [2]. In particular, the recent report published by the Health Insurance Review and Assessment Service Korea in 2018 stated that the health insurance benefits incurred by osteoporosis increased by approximately 43% between 2013 and 2017, and the number of patients that received medical care for the disease during the same period rose by 13%; as more people develop osteoporosis with aging population, it is raised as a serious public health issue not only in advanced countries but also in Korea, which multidimensional studies and measures are being taken at a state-level across the world [3].

Various factors including aging, family history, absorption disorder, underweight, and lack of calcium intake are suggested as risk factors for osteoporosis, but the most important factor is not being able to reach the maximum bone density in the childhood and adolescent period [4]. In short, osteoporosis had been recognized as an adult disease due to aging in the past, but now it is more widely perceived that it begins from the lack of osteogenesis and bone density built in the younger years of life [5].

During the adolescent period, one reaches the maximum bone mass of 90%; for females, the speed of building bone mass reaches the highest at around the age of 13, and after this age, the bone density slightly increases [6,7]. In this period, massive changes in the bone structure take place for physical growth, which leads to rapid osteogenesis and bone remodeling [8]. This lays out the foundation for one to reach the maximum bone mass in life during the adolescent period, however, as the bone mass falls with age, if the maximum bone mass is low in the young years, the risk of having fractures or osteoporosis in the later years of life may increase [9].

The factors that are closely related to the bone mineral density (BMD) include genetic factors, balanced diet, regular physical activities, right BMI and body weight [10,11,12,13,14]. In particular, body weight is the most powerful and positive predictor that imposes physical stress to the bone; osteogenesis and bone density may rise [4,15], but a fall in body weight can lead to bone loss [16]. As such, a positive correlation is well established between body weight and body density in the preceding research, but the influence of the two factors of bodyweight—fat mass (FM) and lean mass (LM)—on the BMD is still undergoing heated discussions [17,18,19,20].

Some of the preceding research found out that fat mass and bone density are in a positive correlation [21,22], but some have also discovered a negative correlation between fat mass and bone parameters [23,24]. Generally, fat mass and lean mass all give a significant impact on bone density, but it is reported that lean mass is more important in the adult group [18].

The research on bone density until today is mainly focused on adults and elderly, especially on females before and after menopause; in particular, in the case of Korea, the research on childhood or on the adolescent period that are critical periods for the accumulation and formation of bone density are insufficiently done. Furthermore, as the factors that enhance bone density in the two periods could be different from those of the adults; more research needs to be done. Therefore, this study assumed that the relationship between lean mass, fat mass, and bone density is different depending on gender; it identifies the body compositions related to the bone density of adolescents.

## 2. Materials and Methods

### 2.1. Design

The Korea National Health and Nutrition Examination Survey (KNHNES) is a nationwide survey on health and nutrition that incorporated the National Health Behavior Survey and National Nutrition Survey that had been carried out independently under article 16 of the National Health Promotion Act enacted in 1995. It has been conducted annually since 2005. People residing in Korea except for foreigners and those in the nursing home, military, or prison are the target population; primary stratification categorizes provinces and cities, and secondary stratification categorizes general regions into 26 strata based on age and population ratio per age group, and apartment-clustered regions into 24 strata based on price per unit space and mean housing size per apartment complex. Within the sample enumeration districts, 20 target households are sampled per district by adopting the systematic sampling method [25,26]. The bone density and body fat examination survey (KNHNES) data used in this study were conducted for those above the age of 19 between July 2008 and June 2009, and those above the age of 10 between July 2009 and May 2011.

### 2.2. Subjects

Subjects were chosen among 21,303 people from the Korea National Health and Nutrition Examination Survey (KNHNES) between 2008 and 2011 that took a bone density checkup; as a result, 1454 teenagers aged between 12 and 18 were selected. The average age of the participants was 15.09 (SE = 0.06); it was 15.13 (SE = 0.08) for males and 15.05 (SE = 0.09) for females, which did not have a statistically significant difference (Wald F = 0.426, *p* = 0.514).

### 2.3. Bone Measurements

The BMD and body composition were measured using the DISCOVERY-W fan-beam densitometer with dual x-ray absorptiometry (DXA; GE Lunar prodigy, Lunar Corporation, Madison, WI, USA) as a standardized method. Additionally, the bone density of femur and lumbar was measured following the bone density examination guideline based on the recommendation of the 2007 International Society for Clinical Densitometry (ISCD) [26].

### 2.4. Analysis Method

As there are differences in fat mass, lean mass, and bone density depending on gender, all the analyses were divided into two genders when conducted. The significance level for the significance test was set at 0.05 for all the analyses, and a weighted complex sample analysis was conducted following the analysis guidance of the Korea National Health and Nutrition Examination Survey (KNHNES). A generalized linear model (GLM) and Rao-Scott Chi-Square analyses were conducted to investigate differences in basic characteristics of each group (fat mass level and lean mass level). As bone density is affected by age, body weight, nutrition (intake of ca, serum vitamin D), walking, muscle strengthening exercises figures were adjusted by body weight, nutrition (intake of ca, serum vitamin D), walking, muscle strengthening exercises, and the bone density differences for lean mass levels were identified with the GLM (generalized linear model) and trend test. Four models were considered to adjust covariates. Model 1 no adjusted variable. Model 2 adjusted for age (year) and area as covariates. Model 3 further adjusted for age (year) and body weight (kg). In model 4, walking and muscle strengthening exercises in addition to model 2. In model 5, nutrition (ca(g) and vitamin D (ng/mL)) in addition to model 3. In model 3, walking (yes/no) and muscle strengthening exercises (yes/no) in addition to model 3. In model 6, walking (yes/no), muscle strengthening exercises (yes/no), and nutrition (ca(g), vitamin D(ng/mL)) in addition to model 3. GLM results were tested by presenting the F value and the *p*-value (*p*) indicating how much the difference was by the fat mass level and lean mass level. The trend test was conducted by presenting a beta (β) and a *p*-value (*p*) of increasing or decreasing bone density as the fat mass level increased. Statistical significance was determined at a level of α=0.05.

## 3. Results

### Analysis of the Bone Density Differences of Fat Mass and Lean Mass Levels

As a result, the difference according to the fat mass in the quintile groups was statistically significant in age, weight, and calcium intake in male adolescents, but only weight in female adolescents (Table 1).

On the other hand, the difference according to the lean mass level was statistically significant in age, weight, and intake of calcium in male adolescents, but statistically significant in age, weight, and muscle strengthening exercises in female adolescents (Table 1).

For male adolescents and female adolescents, the total femur for fat mass in the quintile groups showed a statistically significant increase (male β = 0.027, female β = 0.033) in model 1 and a statistically significant increase (male β = 0.023, female β = 0.030) in model 2. Additionally, for male adolescents the total femur for fat mass in the quintile groups showed a statistically significant decrease (Model 3 β = −0.028, Model 4 β = −0.028, Model 5 β = −0.030, and Model 6 β = −0.030) in model 3−6. However, for female adolescents, a statistically significant difference was not found in model 3–6 (Table 2).

For male adolescents and female adolescents, the total lumbar for fat mass in the quintile groups showed a statistically significant increase (male β = 0.019, female β = 0.016) in model 1 and a statistically significant increase (male β = 0.038, female β = 0.032) in model 2. Additionally, for male adolescents the total femur for fat mass in the quintile groups showed a statistically significant decrease (Model 3 β = −0.034, Model 4 β = −0.034, Model 5 β = −0.035, and Model 6 β = −0.035) in model 3–6. However, for female adolescents, a statistically significant difference was not found in model 3–6 (Table 3).

For male adolescents and female adolescents, the total femur for lean mass in the quintile groups showed a statistically significant increase (male β = 0.064, female β = 0.043) in model 1 and a statistically significant increase (male β = 0.065, female β = 0.036) in model 2. Additionally, for male adolescents the total femur for fat mass in the quintile groups showed a statistically significant decrease (Model 3 β = 0.052, Model 4 β = 0.053, Model 5 β = 0.054, and Model 6 β = 0.054) in model 3–6. However, for female adolescents, a statistically significant difference was found in model 3–4 (Model 3 β = 0.015, Model 4 β = 0.014), but a statistically significant difference was found in model 5–6 adding nutrition as adjustment variable (Table 4).

For male adolescents and female adolescents, the total lumbar for lean mass in the quintile groups showed a statistically significant increase (male β = 0.073, female β = 0.043) in model 1. Moreover, the total lumbar for lean mass in the quintile groups showed a statistically significant increase in model 2 (male β = 0.058, female β = 0.038), 3 (male β = 0.056, female β = 0.019), 4 (male β = 0.057, female β = 0.021), and 5 (male β = 0.057, female β = 0.020; Table 5).

## 4. Discussion

Body composition assessment of adolescents is an integral element for the evaluation of health risk factors, physical strength, and athletic performance along with body size and biological maturity [27,28]. Bodyweight, in particular, is a predictor for bone density; an increase in the body weight leads to more pressure, which stimulates bone formation and increases secretion of estrogen hormone by fat tissues, preventing the osteolysis of osteoclast [29]. Even though being overweight and obesity are the causes that increase the risk of developing metabolic diseases, they have been perceived as positive factors for bone density [30]. The study found out that in regards to fat mass, the bone density of male adolescents significantly decreased when the body fat mass increased, but no significant difference was found for females. Such a result is in line with a report on girls, female teenagers, and young adults in Canada and Mexico that stated body fat is in a negative correlation with the BMD [31,32], with the Nagasaki research that stated body fat mass, lean mass, and BMD are in negative relations among obese girls aged 12 or more and those between 12 and 15 [21], and with a report that found out overweight and obesity of adolescents reduce bone density [33,34,35,36,37,38]. More notably, it was in parallel with other research that identified the correlation between body fat and bone mass after adjusting body weight [39,40]. Chang [33] analyzed how child obesity affects bone density, and it was found that even though body weight gain due to obesity influences mineral content in bones, the secondary hormonal changes and decreased physical movements can rather hamper the formation of mineral in bones.

In conclusion, body fat and body mass index (BMI) have been recognized, in the past, as protective factors of osteoporosis and bone fracture [21,22], however, when BMI reaches over 30 kg/m^2^, it has a negative correlation with bone density [41]. The previous studies [33,34,35,36,37,38] of which the obesity indexes such as the body fat percentage and waist measurement indicate the negative linear relationship with bone density, when the body fat is controlled, in other words, when the weight is identical coincide with the results of this study showing negative correlation of body fat of adolescents with bone density.

In short, such a result implies that to enhance bone density in the teenage years and thereby to prevent osteoporosis, other body compositions need to be more focused rather than on increasing body fat. Additionally, it shows that right body weight management in the adolescent period is helpful to healthy bone density.

In regards to lean mass, it was found that it has a positive impact on the bone density of both male and female adolescents. When the bodyweight was adjusted, it was shown that the increased lean fat leads to a rise in bone density of the two groups. In short, lean mass has a positive correlation with bone density; this is in line with reports of Young et al. [42] that stated the lean mass had a greater impact than fat mass on the increase of bone density in the adolescent years and with other preceding research that reported that lean mass was a significant factor that decides the bone density [43,44,45,46]. Furthermore, according to the Kim and Choi’s [47] study result that compared and analyzed physical development of different bone density levels with female middle school students said that the body mass index, muscle mass, and lean mass were significantly high as the bone density is higher than the expected level for the specific age group on the basis of the Z value (standard deviation score), and also stated that body fat mass and lean mass did have differences but were not significant; it implies that muscle mass and lean mass are critical body compositions to reach maximum bone density.

Meanwhile, the reason that only female adolescents’ lumbar spine showed a significant difference might have been because of the metabolism rate of the spongy bone and the cortical bone in the body. The turnover rate of the spongy bone is faster than the cortical bone [48] the bone metabolism rate of the lumbar spine is higher than that of the femur, as it is rich in spongy bones [49]. Additionally, while the femoral bone density steadily increases over a relatively extended time compared to the lumbar bone density, the lumbar bone density rapidly grows in a short period in the later years [50]. Moreover, as male adolescents build up bone mass faster than their female counterparts [50], the total femur of female adolescents did not show a significant difference in this study.

The influence of muscle mass on bone density is partially determined by an increase in muscular strength [51]; Klein et al. [52] stated that the mass and strength of the upper extremity muscle is an important factor that decides the mass of cortical bones in the upper arm, and Garn et al. [53] reported that the decline in muscle mass is a reason for bone density loss for males. Hughes et al. [54] studied the relationship between muscle mass, muscular strength, and bone density of women, in which they proved the characteristic relation in regards to the bone density in the females’ ward’s triangle. Dolye et al. [54] reported that waist muscles and bone density of the spine have a positive correlation. However, as this study was conducted on deceased subjects in a static posture over an extended time, highly serious muscle loss and static posture have incurred a bone loss. Nonetheless, it is assumed that these results show that the muscles additionally give strain to the bone junctions under the ‘mechanostat’ theory, increasing the bone density [55]. According to this theory, if one raises the maximum muscular strength in the growing period, the mass, size, and strength of the bones improve; physical activities play an important role in maximizing the bone mass in this period [56]. However, even though the correlative mechanism between the physical composition and the BMD has not been verified yet [57,58], the positive effect of lean mass on the bone density is underlined regardless of gender [59,60]. Similar to what Baptista et al. [61] reported, this study could identify that the lean mass of adolescents is an important predictor for bone density.

## 5. Conclusions

During the teenage years in which the bone density and physical growth take place rapidly the elements that enhance bone density are different from those of the adults; therefore, the study took a closer look at how fat mass and lean mass affect bone density by utilizing the survey results of Korea National Health and Nutrition Examination Survey (KNHNES) conducted between 2008 and 2011 on Korean adolescents.

The result of Model 6 considering all correction variables is as follows; in the case of male adolescents, the total femur and lumbar spine showed a significant difference (F = 13.120, *p* < 0.001; F = 12.900, *p* < 0.001) for fat mass, and the trend test showed that the figures significantly decreased (β = −0.030, *p* < 0.001; −0.035, *p* < 0.001). Meanwhile, for lean mass, the total femur and lumbar spine had a significant difference (F = 16.740, *p* < 0.001; F = 20.590, *p* < 0.001) too, but the trend test showed a significant increase (β = 0.054, *p* < 0.001; 0.057, *p* < 0.001). In the case of female adolescents, the lumbar spine (F = 3.600, *p* < 0.05) for lean mass showed a significant difference, and it also significantly rose in the trend test too (β = 0.020, *p* < 0.01).

In short, to strengthen bone density during adolescence, it is important to well manage body fat and improve lean mass in the body. To bring up lean mass in the body, it is considered that conducting a resistive exercise that gives direct stimulus to the bones is important. Additionally, as the high bone density formed during the adolescent period may prevent developing osteoporosis in the old stage of life, well-formed bone density is a highly important factor for the health in the later years of life and also for financial reasons.

It is considered that further prospective research is needed to evaluate the effects of lean mass and fat mass on the BMD of adolescents as this study is a cross-sectional study using survey results of the Korea National Health and Nutrition Examination Survey (KNHNES). Furthermore, further research on whether the increase of lean mass through exercising can directly affect the improvement of bone density needs to be carried out.

## Figures and Tables

**Table 1:** Basic characteristics of fat mass levels and lean mass levels.

		Variable	Q1	Q2	Q3	Q4	Q5	F/χ^2^	*p*
			Mean ± SE	Mean ± SE	Mean ± SE	Mean ± SE	Mean ± SE		
Fat mass	Male	Age (year)	14.79 ± 0.17	15.05 ± 0.2	15.26 ± 0.18	15.3 ± 0.22	15.03 ± 0.19	86.900	<0.0001
		Body weight (kg)	48.94 ± 0.88	53.4 ± 0.77	58.67 ± 0.95	66.03 ± 0.73	77.12 ± 1.12	343.170	<0.0001
		HE_vitD (ng/mL)	16.13 ± 0.69	16.57 ± 0.58	17.46 ± 0.64	16.58 ± 0.63	16.1 ± 0.54	1.820	0.123
		Intake Ca (g)	499.74 ± 30.72	581.74 ± 46.19	567.84 ± 41.48	531.5 ± 32.1	529.6 ± 34.54	2.550	0.038
		Walking (y/*n*)	63/80 (60.2) ^(1)^	56/81 (59.8)	58/80 (59.2)	56/68 (55.5)	60/74 (61.3)	0.840 ^(2)^	0.933
		Muscle strengthening exercises (y/*n*)	90/53 (38.7)	86/51 (41.9)	85/53 (43.8)	84/40 (29.8)	86/48 (38.8)	4.048	0.400
	Female	Age (year)	13.78 ± 0.18	15.08 ± 0.22	15.44 ± 0.24	15.17 ± 0.18	15.48 ± 0.19	1.140	0.337
		Body weight (kg)	42.45 ± 0.45	47.63 ± 0.38	51.1 ± 0.48	56.8 ± 0.45	67.81 ± 1.15	146.820	<0.0001
		HE_vitD (ng/mL)	17.47 ± 0.84	15.35 ± 0.62	15.96 ± 0.62	15.35 ± 0.49	14.87 ± 0.67	0.820	0.513
		Intake Ca (g)	475.29 ± 53.52	422.31 ± 25.38	407.23 ± 21.57	400.89 ± 26.69	428.05 ± 23.99	0.760	0.549
		Walking (y/*n*)	57/62 (53.2)	56/62 (55.3)	60/55 (46.1)	53/62 (58.1)	59/63 (52)	2.576	0.631
		Muscle strengthening exercises (y/*n*)	105/14 (10.9)	113/6 (4.2)	102/13 (13)	100/15 (14.7)	98/24 (16.9)	7.893	0.096
Lean mass	Male	Age (year)	13.01 ± 0.15	14.28 ± 0.19	15.54 ± 0.15	15.99 ± 0.15	16.11 ± 0.12	5.130	0.000
		Body weight (kg)	42.97 ± 0.56	53.77 ± 0.61	59.27 ± 0.6	65.84 ± 0.63	78.12 ± 0.92	147.750	<0.0001
		HE_vitD (ng/mL)	17.28 ± 0.56	17.45 ± 0.64	16.44 ± 0.53	16.1 ± 0.59	15.95 ± 0.51	1.050	0.381
		Intake Ca (g)	469.58 ± 26.12	582.68 ± 34.66	532.22 ± 39.24	528.83 ± 40.13	593.14 ± 44.07	0.380	0.825
		Walking (y/*n*)	70/78 (57.7)	61/77 (58.6)	59/77 (59.5)	55/78 (60.1)	48/73 (60.1)	0.153	0.997
		Muscle strengthening exercises (y/*n*)	109/39 (26.2)	90/48 (39.2)	81/55 (41.5)	85/48 (40.2)	66/55 (44.8)	6.713	0.152
	Female	Age (year)	14.37 ± 0.2	14.67 ± 0.21	15.37 ± 0.19	15.33 ± 0.23	15.39 ± 0.2	12.720	<0.0001
		Body weight (kg)	42.97 ± 0.46	47.82 ± 0.39	51.84 ± 0.53	56.67 ± 0.61	67.18 ± 1.17	198.320	<0.0001
		HE_vitD (ng/mL)	16.03 ± 0.54	16.53 ± 0.61	15.91 ± 0.72	14.99 ± 0.67	15.4 ± 0.71	2.260	0.061
		Intake Ca (g)	402.41 ± 20.35	413.58 ± 25.01	420.76 ± 26.48	451.13 ± 43.5	430.81 ± 20.91	0.380	0.820
		Walking (y/*n*)	52/64 (55.6)	69/48 (43.9)	57/62 (54.3)	49/68 (54.7)	58/62 (54.3)	2.980	0.561
		Muscle strengthening exercises (y/*n*)	106/10 (6.6)	113/5 (4.1)	106/13 (9.8)	99/18 (18.1)	94/26 (19.7)	16.018	0.003

^(1)^: of yes/# of no (% of yes). ^(2)^: Rao-Scott Chi-Square.

**Table 2:** Analysis of the bone density (total femur) differences of fat mass levels.

		Fat Mass Group					F	*p*	β	Trend *p*
		Q1	Q2	Q3	Q4	Q5				
Male	Model 1	0.866 ± 0.015 ^(1)^	0.903 ± 0.014	0.934 ± 0.014	0.956 ± 0.012	0.974 ± 0.011	10.760	<0.0001	0.027	<0.0001
	Model 2	0.875 ± 0.015	0.903 ± 0.014	0.931 ± 0.013	0.95 ± 0.012	0.977 ± 0.01	11.430	<0.0001	0.023	<0.0001
	Model 3	0.972 ± 0.014	0.966 ± 0.013	0.954 ± 0.01	0.912 ± 0.01	0.833 ± 0.016	9.800	<0.0001	−0.028	<0.0001
	Model 4	0.97 ± 0.014	0.964 ± 0.013	0.951 ± 0.01	0.909 ± 0.011	0.828 ± 0.015	10.920	<0.0001	−0.028	<0.0001
	Model 5	0.975 ± 0.015	0.971 ± 0.014	0.95 ± 0.011	0.915 ± 0.012	0.821 ± 0.016	11.660	<0.0001	−0.030	<0.0001
	Model 6	0.974 ± 0.015	0.968 ± 0.013	0.948 ± 0.011	0.913 ± 0.012	0.816 ± 0.016	13.120	<0.0001	−0.030	<0.0001
Female	Model 1	0.804 ± 0.012	0.849 ± 0.013	0.862 ± 0.012	0.898 ± 0.011	0.944 ± 0.009	25.770	<0.0001	0.033	<0.0001
	Model 2	0.814 ± 0.013	0.849 ± 0.013	0.858 ± 0.012	0.896 ± 0.01	0.941 ± 0.009	22.930	<0.0001	0.030	<0.0001
	Model 3	0.879 ± 0.016	0.884 ± 0.013	0.872 ± 0.011	0.874 ± 0.011	0.853 ± 0.018	0.640	0.636	−0.005	0.469
	Model 4	0.875 ± 0.016	0.886 ± 0.014	0.871 ± 0.012	0.875 ± 0.011	0.856 ± 0.018	0.620	0.648	−0.004	0.559
	Model 5	0.868 ± 0.017	0.894 ± 0.016	0.871 ± 0.013	0.856 ± 0.012	0.853 ± 0.021	1.040	0.384	−0.007	0.393
	Model 6	0.866 ± 0.017	0.894 ± 0.016	0.870 ± 0.013	0.854 ± 0.012	0.854 ± 0.021	1.230	0.295	−0.007	0.395

^(1)^: Mean ± SE. Model 2: Adjusted by age. Model 3: Adjusted by age, weight. Model 4: Adjusted by age, weight, walking, muscle strengthening exercises. Model 5: Adjusted by age, weight, nutrition (intake of ca, serum vitamin D). Model 6: Adjusted by age, weight, walking, muscle strengthening exercises, nutrition (intake of ca, serum vitamin D).

**Table 3:** Analysis of the bone density (total lumbar spine) differences of fat mass levels.

		Fat Mass Group					F	*p*	β	Trend *p*
		Q1	Q2	Q3	Q4	Q5				
Male	Model 1	0.798 ± 0.015 ^(1)^	0.836 ± 0.012	0.852 ± 0.013	0.868 ± 0.013	0.876 ± 0.013	4.520	0.0013	0.019	<0.0001
	Model 2	0.814 ± 0.014	0.834 ± 0.009	0.848 ± 0.012	0.857 ± 0.012	0.882 ± 0.010	5.280	0.0003	0.016	<0.0001
	Model 3	0.906 ± 0.012	0.893 ± 0.009	0.87 ± 0.009	0.824 ± 0.011	0.748 ± 0.014	16.950	<0.0001	−0.034	<0.0001
	Model 4	0.904 ± 0.013	0.891 ± 0.009	0.867 ± 0.009	0.822 ± 0.011	0.746 ± 0.013	17.450	<0.0001	−0.034	<0.0001
	Model 5	0.904 ± 0.012	0.895 ± 0.011	0.860 ± 0.011	0.828 ± 0.01	0.740 ± 0.017	12.900	<0.0001	−0.035	<0.0001
	Model 6	0.903 ± 0.013	0.894 ± 0.011	0.859 ± 0.011	0.827 ± 0.01	0.739 ± 0.017	12.900	<0.0001	−0.035	<0.0001
Female	Model 1	0.802 ± 0.013	0.873 ± 0.012	0.885 ± 0.012	0.921 ± 0.012	0.969 ± 0.012	29.590	<0.0001	0.038	<0.0001
	Model 2	0.826 ± 0.013	0.873 ± 0.012	0.876 ± 0.010	0.918 ± 0.012	0.961 ± 0.011	21.810	<0.0001	0.032	<0.0001
	Model 3	0.886 ± 0.015	0.905 ± 0.013	0.890 ± 0.009	0.898 ± 0.012	0.879 ± 0.016	0.750	0.558	−0.001	0.855
	Model 4	0.881 ± 0.015	0.905 ± 0.012	0.887 ± 0.009	0.898 ± 0.012	0.883 ± 0.016	0.810	0.519	0.000	0.975
	Model 5	0.870 ± 0.017	0.895 ± 0.015	0.884 ± 0.011	0.884 ± 0.011	0.891 ± 0.019	0.450	0.774	0.002	0.744
	Model 6	0.869 ± 0.017	0.895 ± 0.014	0.883 ± 0.011	0.883 ± 0.011	0.894 ± 0.019	0.510	0.726	0.003	0.703

^(1)^: Mean ± SE. Model 2: Adjusted by age. Model 3: Adjusted by age, weight. Model 4: Adjusted by age, weight, walking, muscle strengthening exercises. Model 5: Adjusted by age, weight, nutrition (intake of ca, serum vitamin D). Model 6: Adjusted by age, weight, walking, muscle strengthening exercises, nutrition (intake of ca, serum vitamin D).

**Table 4:** Analysis of the bone density (total femur) differences of lean mass levels.

		Lean Mass Group					F	*p*	β	Trend *p*
		Q1	Q2	Q3	Q4	Q5				
Male	Model 1	0.780 ± 0.008 ^(1)^	0.863 ± 0.010	0.941 ± 0.010	0.980 ± 0.012	1.041 ± 0.012	108.530	<0.0001	0.064	<0.0001
	Model 2	0.774 ± 0.011	0.860 ± 0.011	0.942 ± 0.010	0.983 ± 0.012	1.044 ± 0.012	78.460	<0.0001	0.065	<0.0001
	Model 3	0.802 ± 0.015	0.871 ± 0.011	0.943 ± 0.010	0.975 ± 0.012	1.018 ± 0.017	22.250	<0.0001	0.052	<0.0001
	Model 4	0.798 ± 0.016	0.868 ± 0.012	0.940 ± 0.010	0.972 ± 0.011	1.017 ± 0.017	21.700	<0.0001	0.053	<0.0001
	Model 5	0.798 ± 0.016	0.869 ± 0.013	0.939 ± 0.012	0.966 ± 0.013	1.029 ± 0.019	16.270	<0.0001	0.054	<0.0001
	Model 6	0.794 ± 0.016	0.867 ± 0.014	0.937 ± 0.011	0.965 ± 0.013	1.028 ± 0.018	16.740	<0.0001	0.054	<0.0001
Female	Model 1	0.795 ± 0.012	0.838 ± 0.010	0.875 ± 0.012	0.903 ± 0.010	0.954 ± 0.010	29.290	<0.0001	0.043	<0.0001
	Model 2	0.800 ± 0.013	0.840 ± 0.010	0.874 ± 0.012	0.900 ± 0.010	0.951 ± 0.009	26.130	<0.0001	0.036	<0.0001
	Model 3	0.839 ± 0.016	0.860 ± 0.011	0.880 ± 0.012	0.887 ± 0.011	0.898 ± 0.013	1.440	0.220	0.015	0.020
	Model 4	0.839 ± 0.016	0.863 ± 0.012	0.880 ± 0.012	0.885 ± 0.012	0.895 ± 0.013	1.400	0.232	0.014	0.035
	Model 5	0.846 ± 0.018	0.857 ± 0.010	0.870 ± 0.014	0.885 ± 0.015	0.884 ± 0.015	0.630	0.6424	0.011	0.131
	Model 6	0.846 ± 0.018	0.860 ± 0.010	0.869 ± 0.013	0.883 ± 0.016	0.881 ± 0.015	0.460	0.7659	0.010	0.186

^(1)^: Mean ± SE. Model 2: Adjusted by age. Model 3: Adjusted by age, weight. Model 4: Adjusted by age, weight, walking, muscle strengthening exercises. Model 5: Adjusted by age, weight, nutrition (intake of ca, serum vitamin D). Model 6: Adjusted by age, weight, walking, muscle strengthening exercises, nutrition (intake of ca, serum vitamin D).

**Table 5:** Analysis of the bone density (total lumbar spine) differences of lean mass levels.

		Lean Mass Group					F	*p*	β	Trend *p*
		Q1	Q2	Q3	Q4	Q5				
Male	Model 1	0.668 ± 0.011 ^(1)^	0.777 ± 0.010	0.868 ± 0.008	0.930 ± 0.009	0.956 ± 0.011	101.780	<0.0001	0.073	<0.0001
	Model 2	0.703 ± 0.012	0.790 ± 0.010	0.862 ± 0.007	0.914 ± 0.009	0.939 ± 0.011	60.710	<0.0001	0.058	<0.0001
	Model 3	0.708 ± 0.014	0.792 ± 0.010	0.862 ± 0.007	0.913 ± 0.009	0.934 ± 0.014	33.570	<0.0001	0.056	<0.0001
	Model 4	0.708 ± 0.015	0.790 ± 0.010	0.861 ± 0.008	0.910 ± 0.009	0.931 ± 0.014	31.470	<0.0001	0.056	<0.0001
	Model 5	0.702 ± 0.016	0.788 ± 0.015	0.862 ± 0.009	0.899 ± 0.009	0.940 ± 0.016	21.460	<0.0001	0.057	<0.0001
	Model 6	0.702 ± 0.016	0.788 ± 0.015	0.862 ± 0.009	0.898 ± 0.010	0.939 ± 0.016	20.590	<0.0001	0.057	<0.0001
Female	Model 1	0.800 ± 0.012	0.851 ± 0.008	0.905 ± 0.011	0.923 ± 0.011	0.981 ± 0.012	34.050	<0.0001	0.043	<0.0001
	Model 2	0.813 ± 0.011	0.858 ± 0.008	0.901 ± 0.011	0.917 ± 0.010	0.973 ± 0.011	30.670	<0.0001	0.038	<0.0001
	Model 3	0.846 ± 0.014	0.875 ± 0.009	0.906 ± 0.011	0.906 ± 0.011	0.926 ± 0.015	3.200	0.0128	0.020	0.001
	Model 4	0.846 ± 0.014	0.879 ± 0.009	0.905 ± 0.011	0.903 ± 0.011	0.923 ± 0.015	3.270	0.0114	0.019	0.002
	Model 5	0.833 ± 0.016	0.871 ± 0.010	0.897 ± 0.013	0.901 ± 0.013	0.918 ± 0.015	2.750	0.0274	0.021	0.003
	Model 6	0.834 ± 0.016	0.874 ± 0.010	0.896 ± 0.013	0.899 ± 0.013	0.915 ± 0.015	2.600	0.0353	0.020	0.005

^(1)^: Mean ± SE. Model 2: Adjusted by age. Model 3: Adjusted by age, weight. Model 4: Adjusted by age, weight, walking, muscle strengthening exercises. Model 5: Adjusted by age, weight, nutrition (intake of ca, serum vitamin D). Model 6: Adjusted by age, weight, walking, muscle strengthening exercises, nutrition (intake of ca, serum vitamin D).
